# Supplementary figures and images for: Twitching motility suppressors reveal a role for FimX in type IV pilus extension dynamics
Source: PLoS Genet. 2025 Oct 13;21(10):e1011802. doi: 10.1371/journal.pgen.1011802 (PMC12533971; doi:10.1371/journal.pgen.1011802)

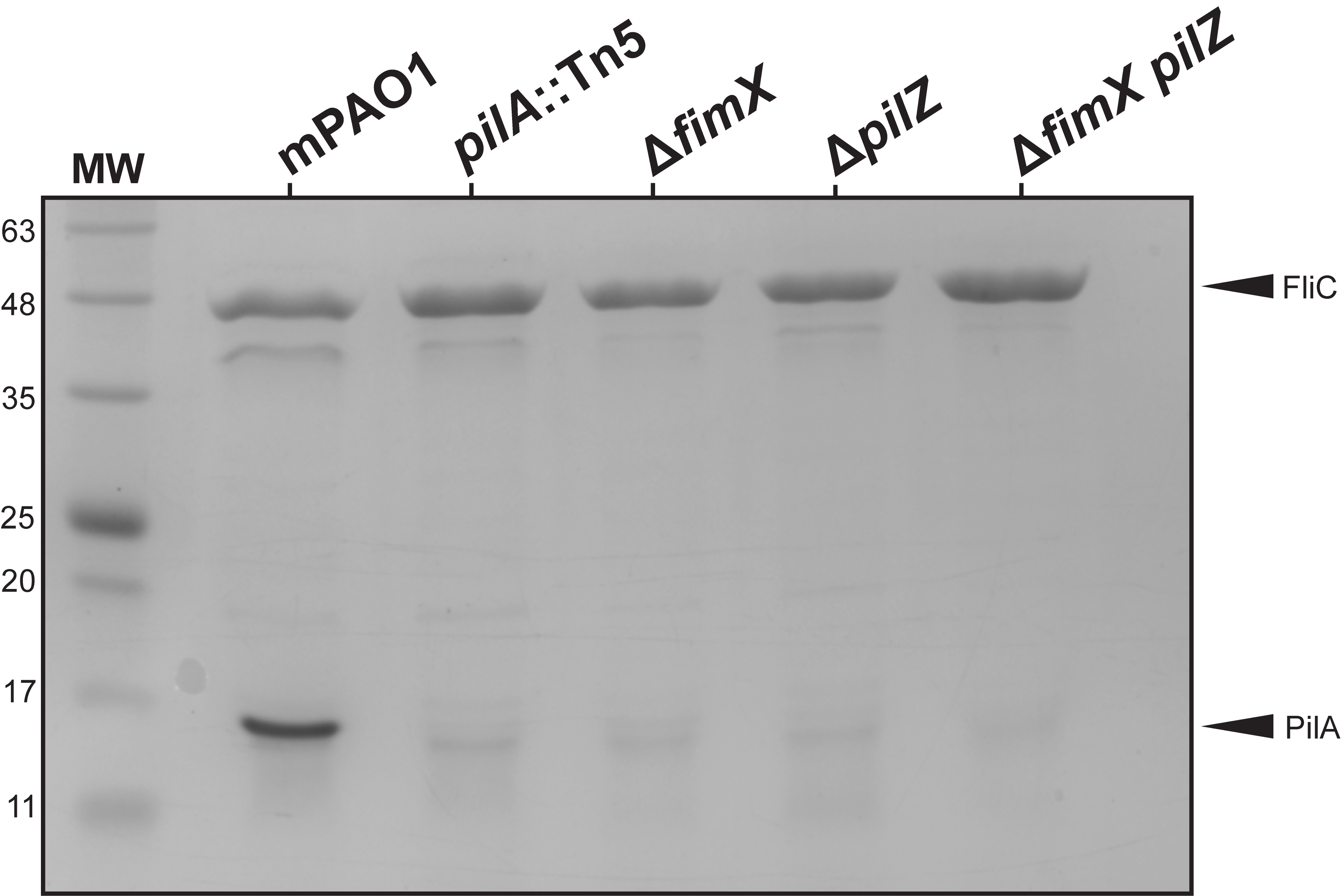

Supplement: S1 Fig — Representative SDS-PAGE of sheared surface proteins. Major pilin subunits (PilA) are indicated. Sample loading was normalized to flagellin (FliC) levels. (TIF) [file pgen.1011802.s001.tif]

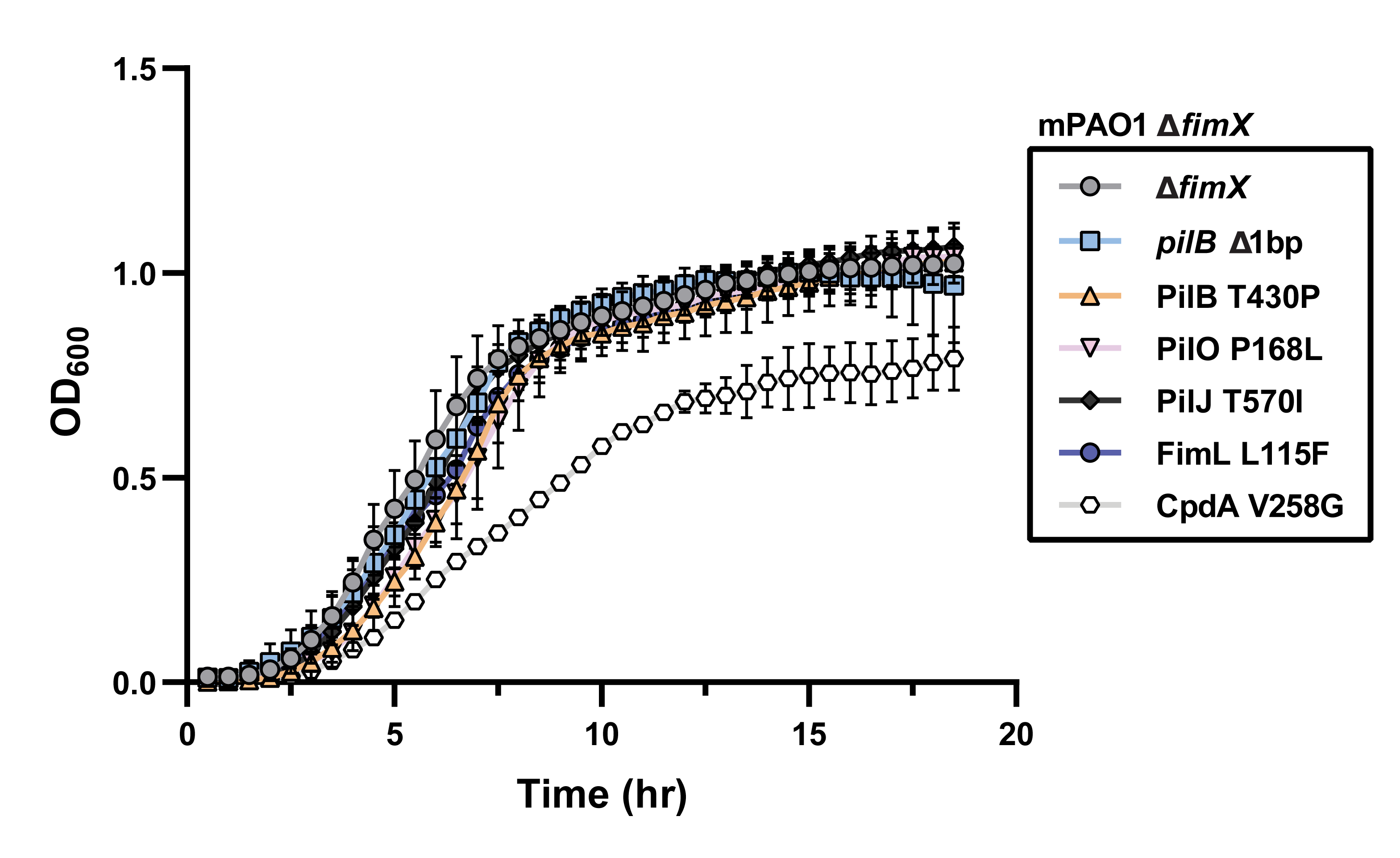

Supplement: S2 Fig — Bacterial growth curves across 18 hours in LB media. Points represent the means of triplicate samples from three independent experiments ± SD. (TIF) [file pgen.1011802.s002.tif]

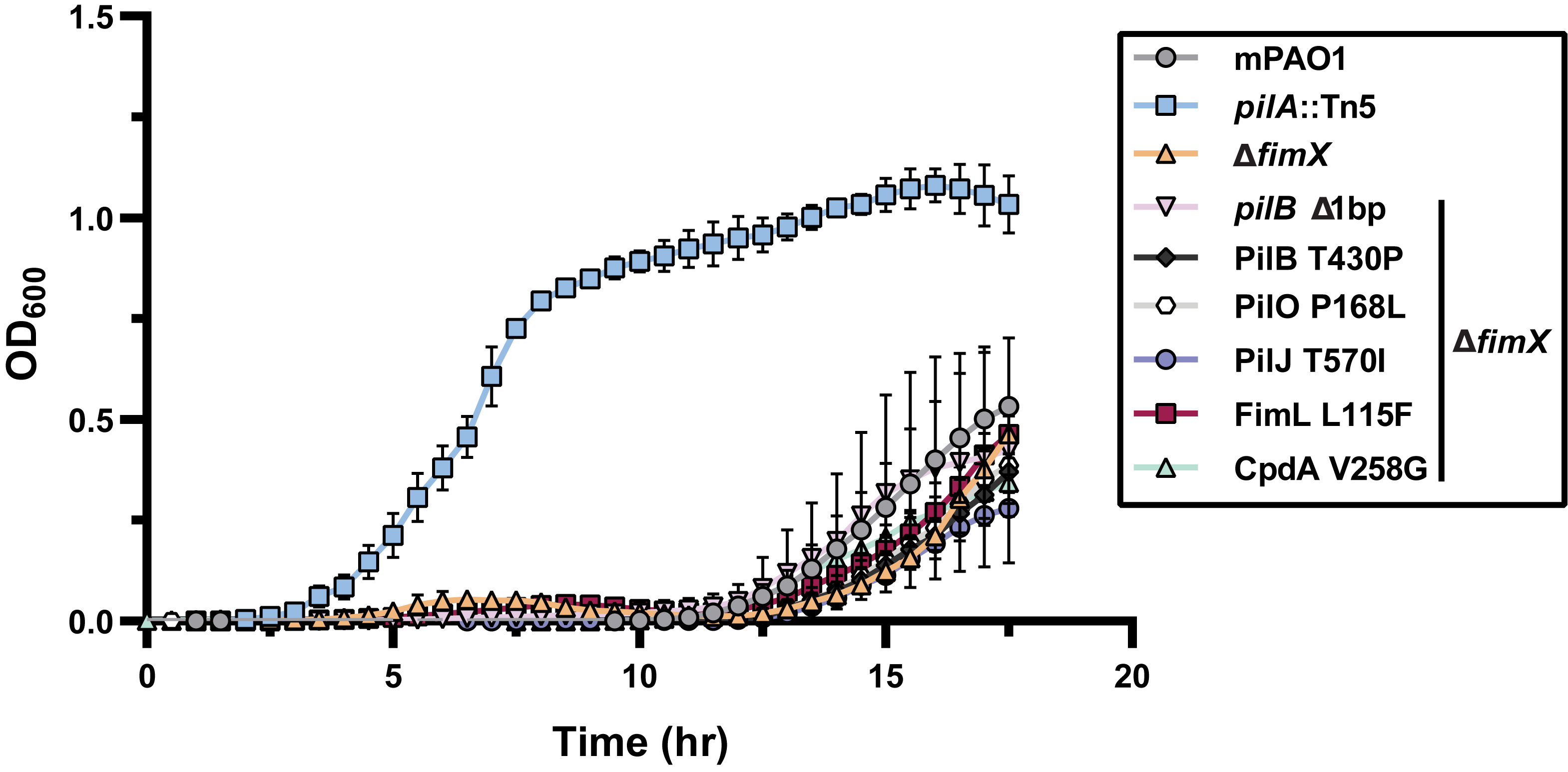

Supplement: S3 Fig — Bacterial growth curves across 18 hours in LB media with PO4 phage-challenge. Points represent the means of triplicate samples from three independent experiments ± SD. (TIF) [file pgen.1011802.s003.tif]

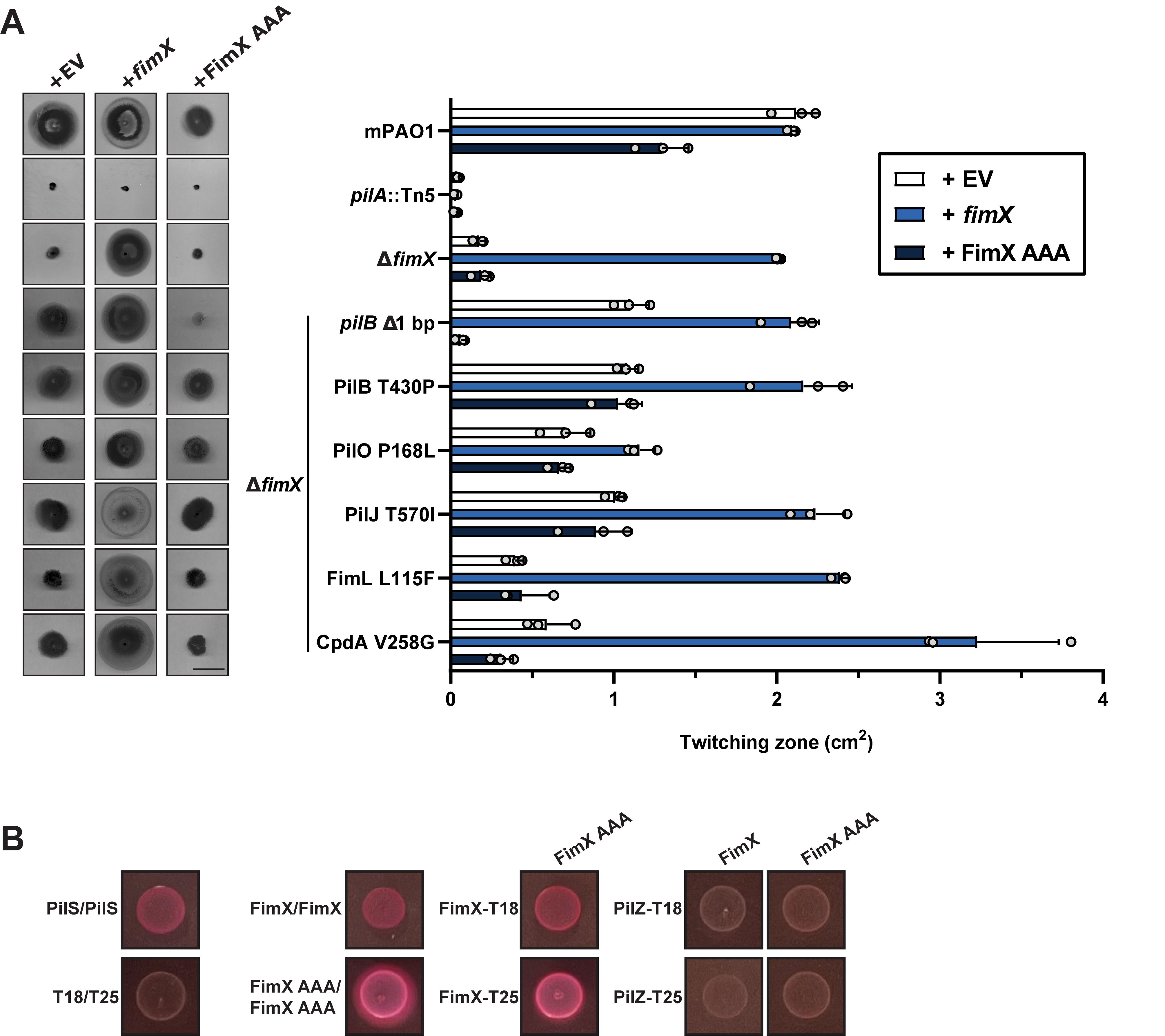

Supplement: S4 Fig — (A) Quantification of sub-agar stab twitching motility zones for ΔfimX twitching suppressor mutants complemented with FimX variants. Representative crystal violet-stained twitching zones are shown to the left. Scale bar = 1 cm. Bars represent the means of triplicate samples from three independent experiments ± SD. (B) Representative colonies showing pairwise interactions (pink) between FimX and FimX AAA mutant. PilZ was used as a known non-interacting negative control with FimX [56]. Untagged T18/T25 plasmid and PilS-T18/PilS-T25 homodimers [69] were used as negative and positive controls respectively. EV: empty pHERD30T vector, fimX: fimX in pHERD30T vector, FimX AAA: FimX AAA in pHERD30T vector. (TIF) [file pgen.1011802.s004.tif]

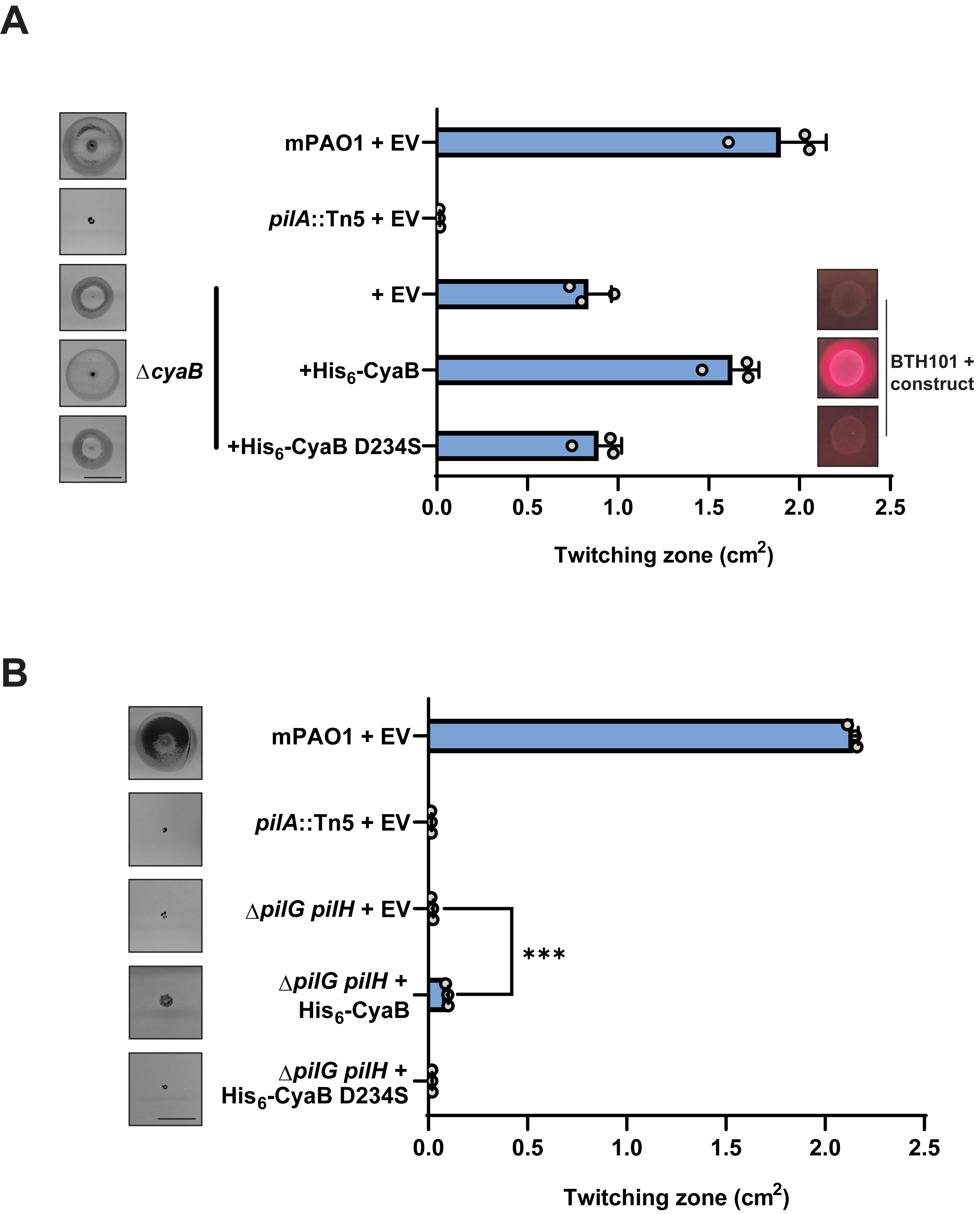

Supplement: S5 Fig — (A) Quantification of sub-agar stab twitching motility zones for a ΔcyaB mutant complemented with CyaB or a catalytically inactive mutant (D234S). Representative CyaB or CyaB D234S-expressing BTH101 colonies are shown in the inset to the right. Pink colour results from increased cAMP levels. Representative crystal violet-stained twitching zones are shown to the left. Bars represent the means of triplicate samples from three independent experiments ± SD. (B) Quantification of sub-agar stab twitching motility zones for a ΔpilGH double mutant complemented with CyaB. Representative twitching zones are shown to the left. Bars represent the means of triplicate samples from three independent experiments ± SD. All scale bars = 1 cm. ***: p ≤ 0.001 (Two-tailed Welch’s t-test). EV: empty pHERD30T vector, His6-CyaB (D234S): N-terminally hexa-histidine tagged CyaB or CyaB D234S in pHERD30T vector. (TIF) [file pgen.1011802.s005.tif]

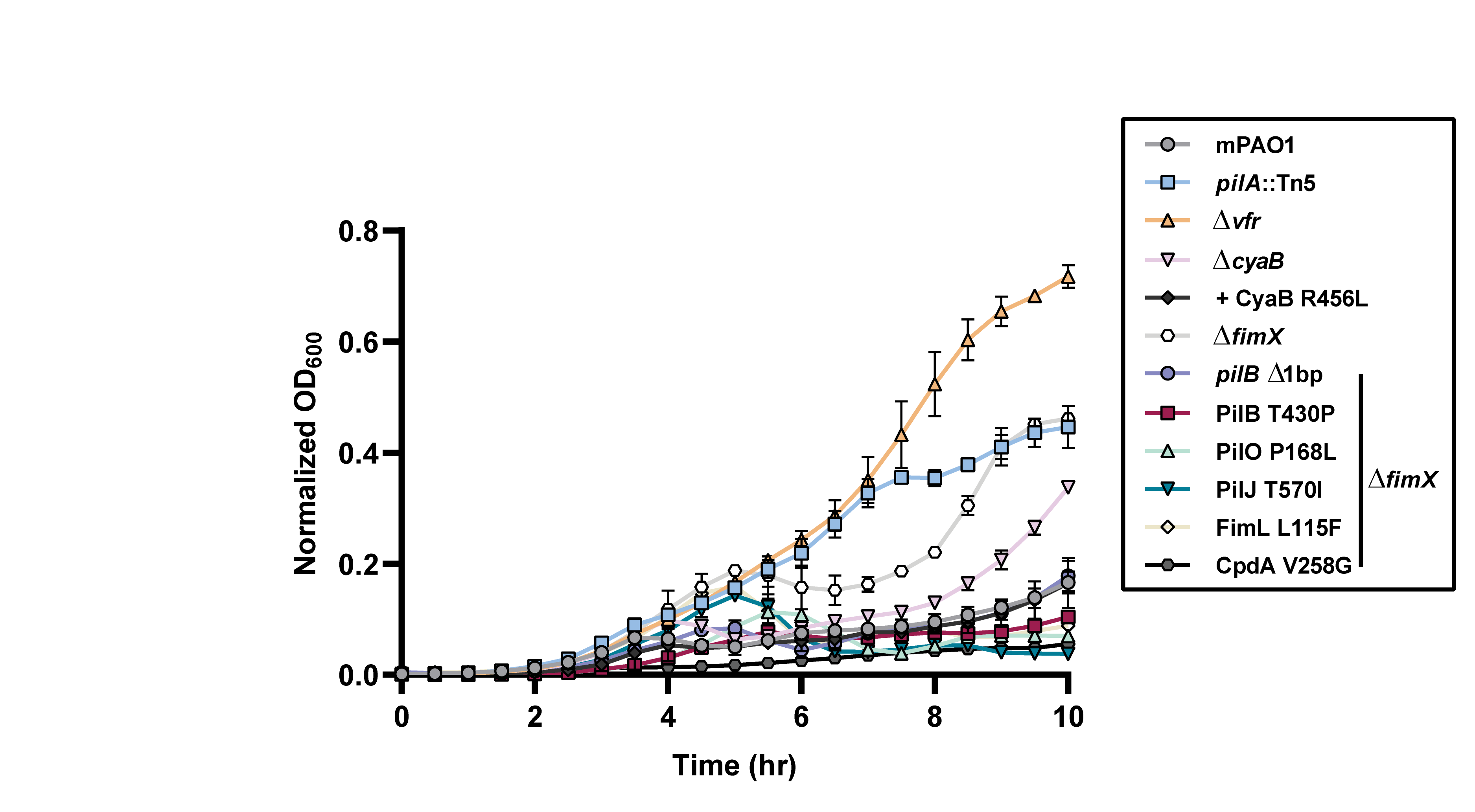

Supplement: S6 Fig — Bacterial growth curves across 10 hours in LB media with PO4 phage-challenge. Points represent the means of triplicate samples from two independent experiments ± SD. CyaB R456L: CyaB R456L in pBADGr vector. (TIF) [file pgen.1011802.s006.tif]

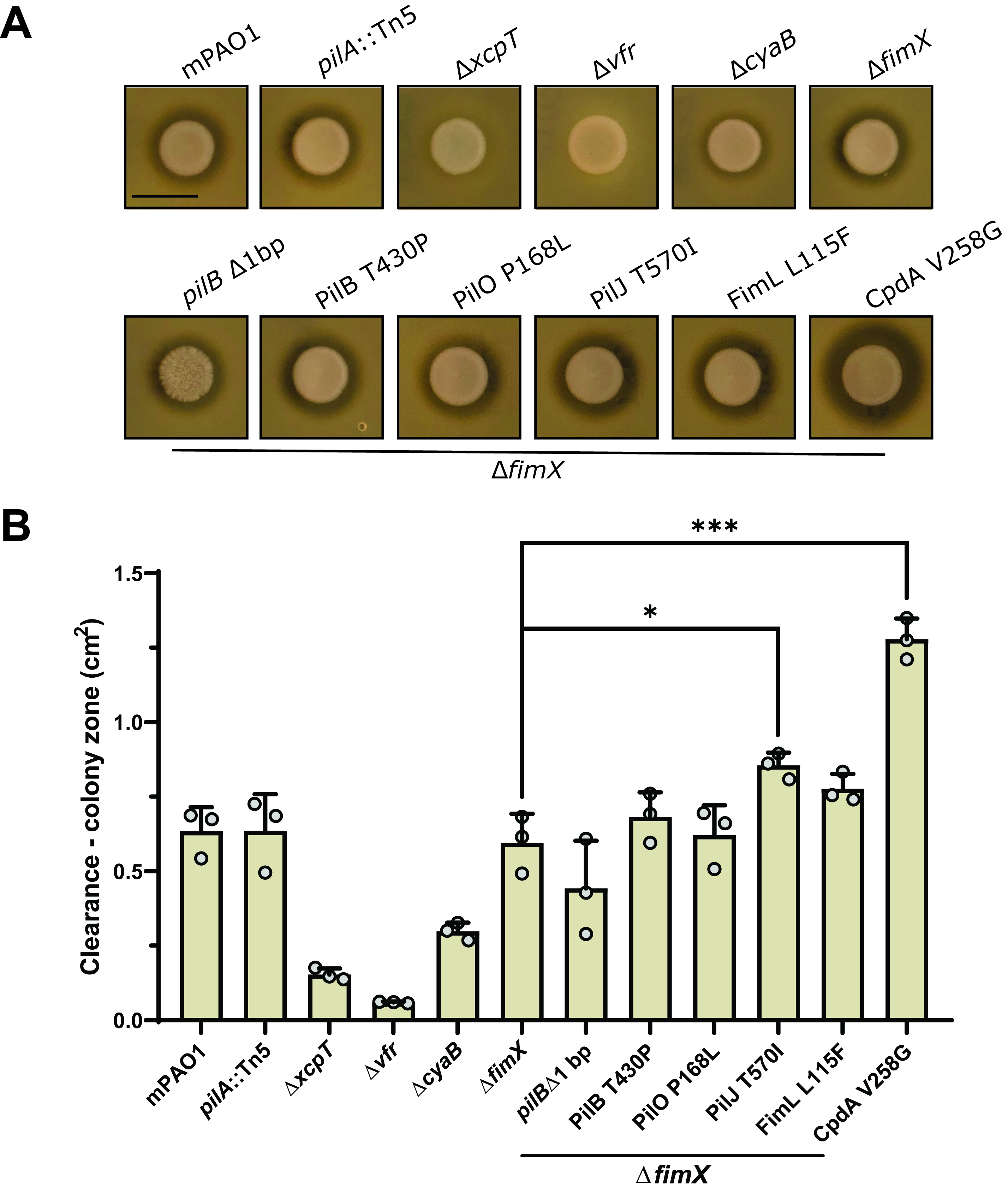

Supplement: S7 Fig — (A) Representative colonies and surrounding zones of clearance for the ΔfimX twitching suppressor mutants. Scale bar = 1 cm. (B) Quantification of the colony area subtracted from the skim milk clearance area. Bars represent the means of duplicate samples from three independent experiments ± SD. *: 0.05 ≥ p ≥ 0.01; ***: 0.001 ≥ p (One-way ANOVA). (TIF) [file pgen.1011802.s007.tif]

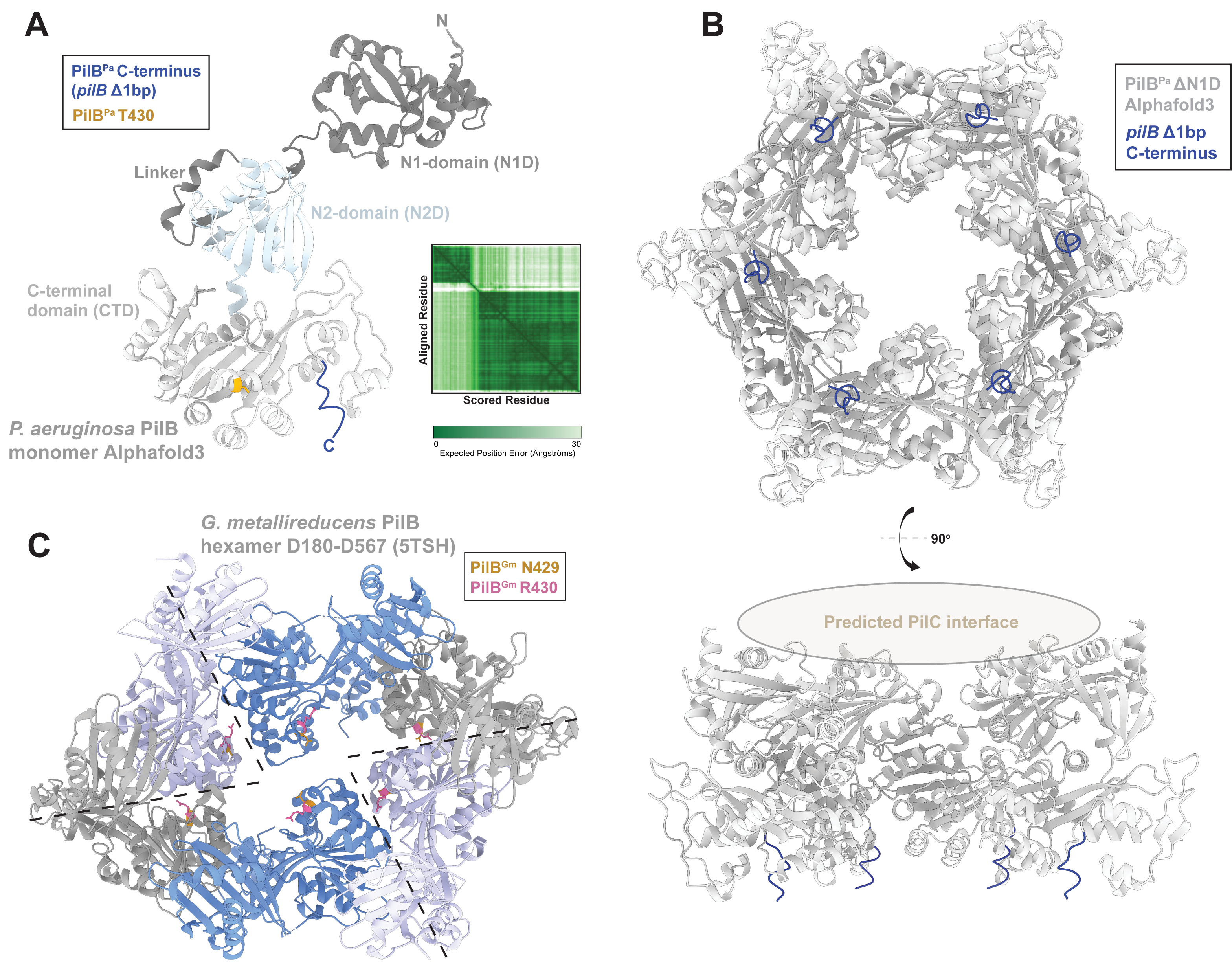

Supplement: S8 Fig — The N1D and linker region (dark grey), N2D (light blue), and CTD (white) are indicated. The pilB Δ1bp C-terminal extension is shown in dark blue. PilB T430 side chain is shown in stick and in orange. The model PAE plot is shown to the right. (B) pilB Δ1bp full length homohexameric Alphafold3 predicted model is shown below with the extended sequence residues highligted in dark blue. The predicted PilC interface is shown on the bottom. The N1Ds and linker regions for each monomer are hidden. The model on the bottom has the two front monomers hidden for clarity. (C) X-ray crystal structure of hexameric PilB from G. metallireducens (5TSH) with sequence aligned residues of interest N429 (PilBPa T430) and R430 (PilBPa R431) highlighted in orange and magenta, respectively. C2-symetric monmers are highlited in the same colours. Dashed lines indicate approximate protomer interfaces. (TIF) [file pgen.1011802.s008.tif]

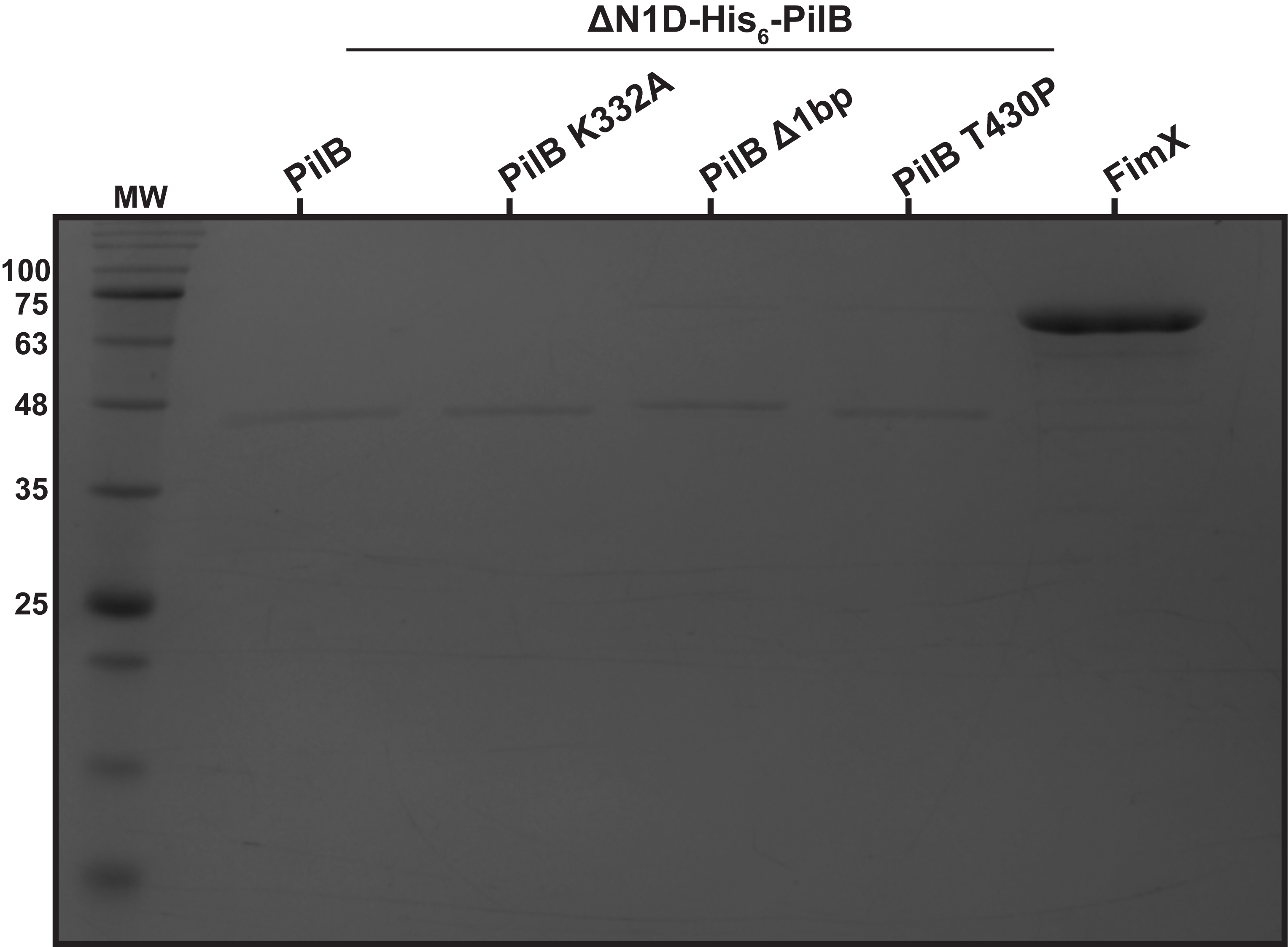

Supplement: S12 Fig — SDS-PAGE of representative protein samples used in the study. Samples are diluted to the same concentrations added to the reaction mixture preparation. (TIF) [file pgen.1011802.s012.tif]

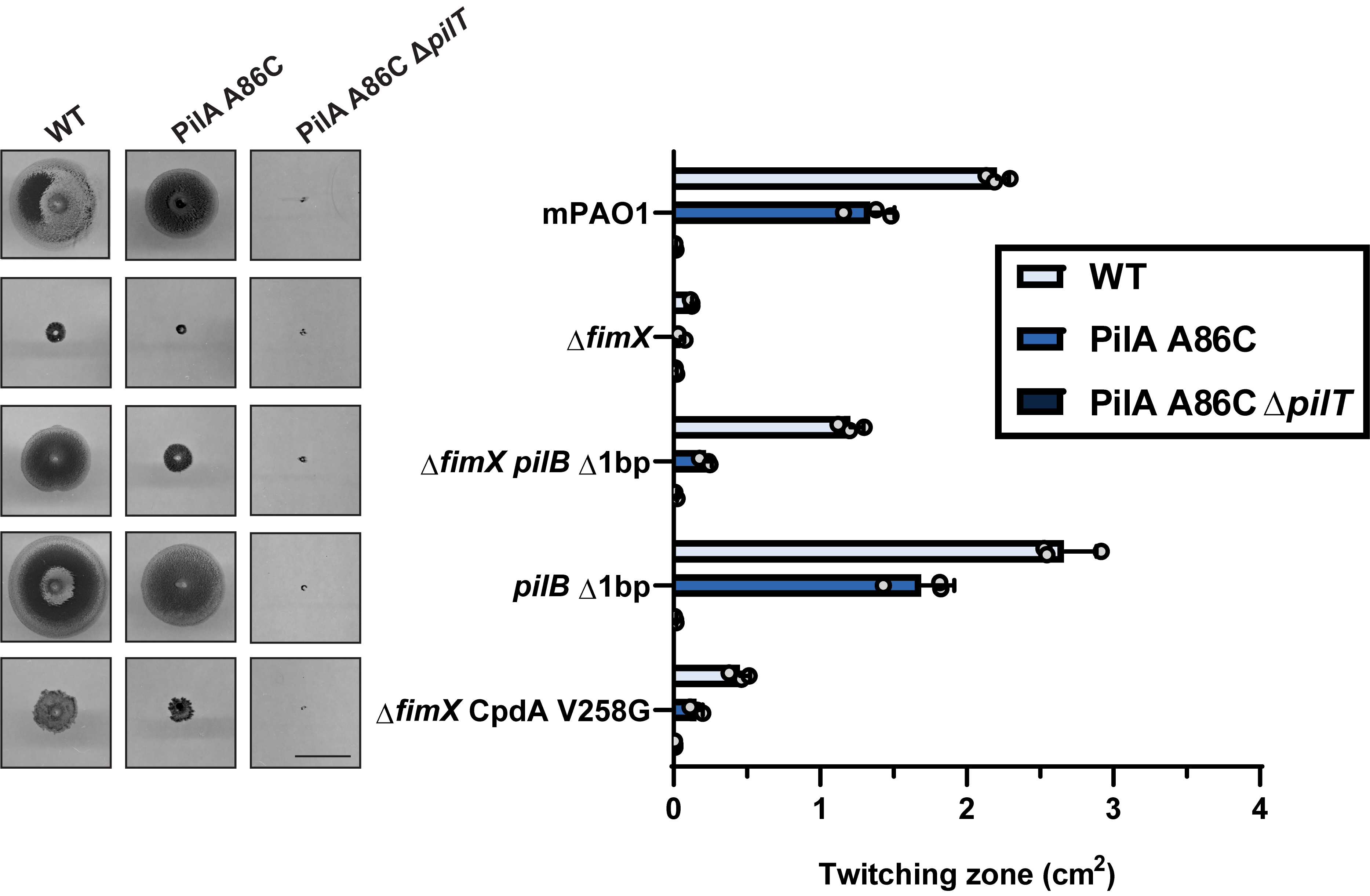

Supplement: S13 Fig — Quantification of sub-agar stab twitching zone areas of PilA A86C mutants. Representative crystal violet stained twitching zones are shown to the left. Bars represent the means of triplicate samples from three independent experiments ± SD. Scale bar = 1 cm. (TIF) [file pgen.1011802.s013.tif]

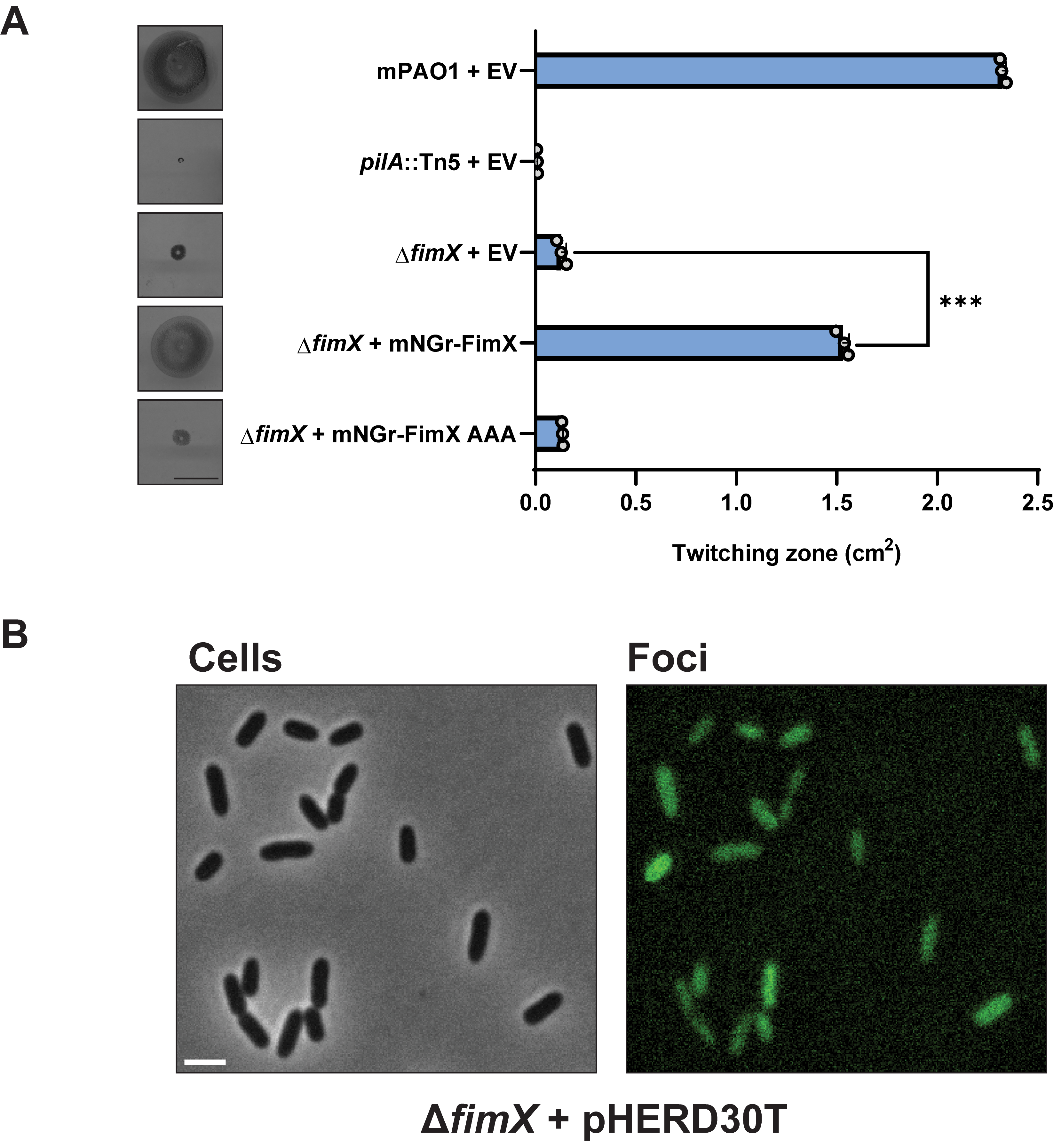

Supplement: S14 Fig — (A) Quantification of sub-agar stab twitching zone areas of ΔfimX mutants complemented with N-terminal fusion of mNeonGreen (mNGr) to FimX. Representative crystal violet stained twitching zones are shown to the left. Expression was not induced with arabinose. Bars represent the means of triplicate samples from three independent experiments ± SD. Scale bar = 1 cm. EV: empty pHERD30T, mNGr-FimX (AAA): N-terminal fusion of mNeonGreen to FimX or FimX AAA in pHERD30T vector. (B) Representative images of ΔfimX mutant cells carrying empty pHERD30T with background fluorescence subtracted. Scale bar = 2 µm. ***: 0.001 ≥ p (Two-tailed Welch’s t-test). (TIF) [file pgen.1011802.s014.tif]

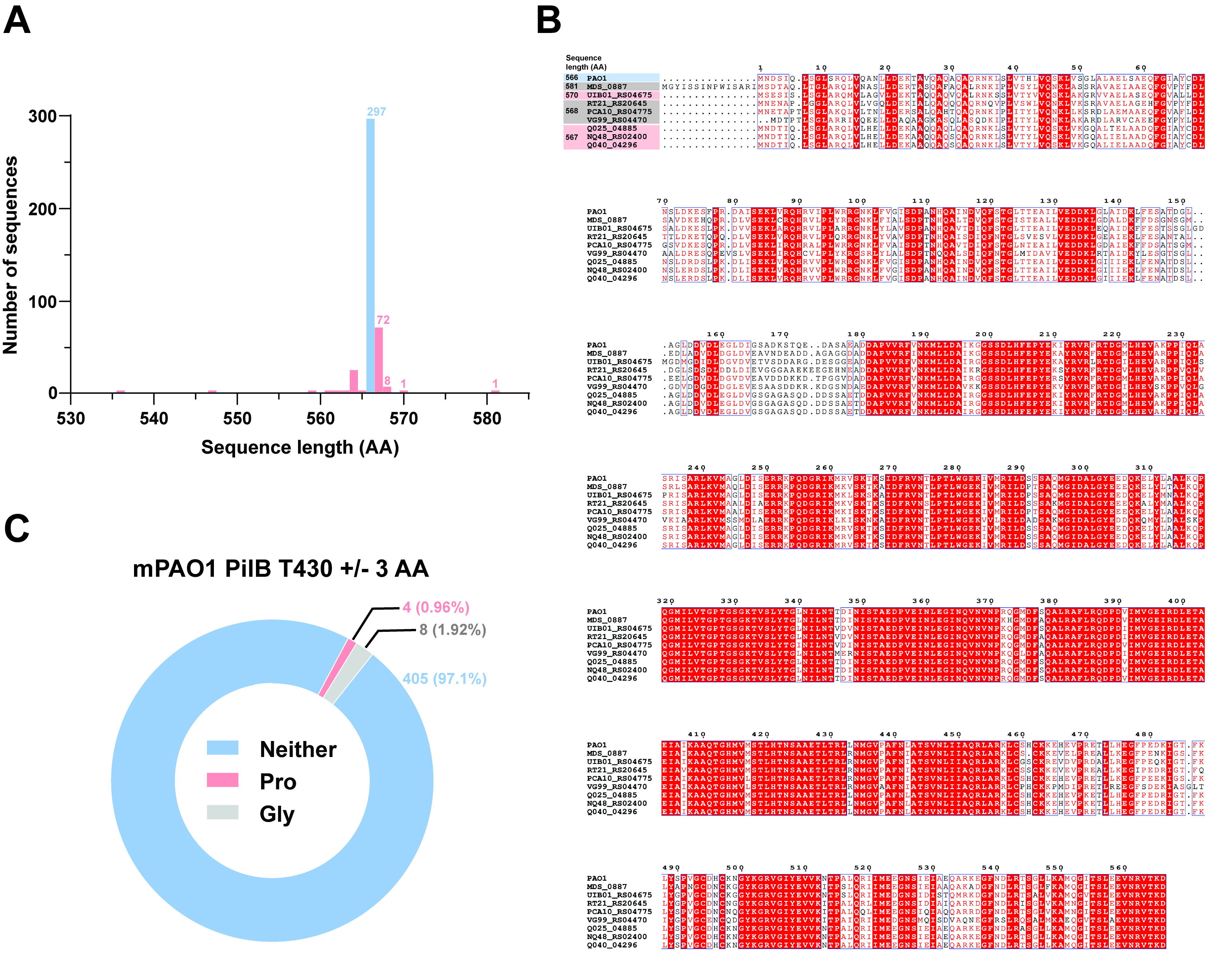

Supplement: S15 Fig — (A) Total protein sequence length of PilB orthologues in P. aeruginosa from the Pseudomonas.com database. Sequences which are the same length as PAO1 PilB (566 residues) are indicated in blue. (B) Full length sequence alignment of representative P. aeruginosa orthologues of varying length. Sequence lengths are indicated in the boxes to the left. Identical residues are highlighted in red, and regions of sequence similarity are grouped in boxes. (C) Percentage of sequences with an identifiable α-helix-disrupting residue ± three amino acids from the position which aligns with PAO1 PilB T430. (TIF) [file pgen.1011802.s015.tif]
