# Supplementary material for: Twitching motility suppressors reveal a role for FimX in type IV pilus extension dynamics
Source: PLoS Genet. 2025 Oct 13;21(10):e1011802. doi: 10.1371/journal.pgen.1011802 (PMC12533971; doi:10.1371/journal.pgen.1011802)
Supplement: S1 Table — (DOCX) [file pgen.1011802.s016.docx]

**Table S1: All putative suppressor mutations identified in this study.**

| **Gene** | **Mutation** | **Function** | **Validated?** |
| --- | --- | --- | --- |
| **PilB** | | | |
| *pilB* | Δ1bp – A1697 | Extension ATPase | Y |
| *pilB* | T430P | - | Y |
| *pilB* | D181N | - | N |
| *pilB* | E558D | - | Y |
| **Pil-Chp network** | | | |
| *pilJ* | T570I | Methyl-accepting chemotaxis protein – transduces signal for cAMP synthesis | Y |
| *fimL* | L115F | Scaffold protein – involved in CyaB activation | Y |
| *chpA* | Δ36bp – 3776-3811 | Histidine kinase – required for cAMP upregulation | N |
| *chpA* | Δ39bp – 3856-3894 | - | N |
| **Other** | | | |
| *pilO* | P168L | Alignment complex structural protein | Y |
| *cpdA* | V258G | cAMP phosphodiesterase | Y |
| *cpdA* | 575-599 sequence duplication AGGTACGCTGCCTGCTGTGGGGGCA | - | N |
| *vfr* | G146R | Transcription factor | N |
