# Supplementary material for: Twitching motility suppressors reveal a role for FimX in type IV pilus extension dynamics
Source: PLoS Genet. 2025 Oct 13;21(10):e1011802. doi: 10.1371/journal.pgen.1011802 (PMC12533971; doi:10.1371/journal.pgen.1011802)
Supplement: S2 Table — (DOCX) [file pgen.1011802.s017.docx]

**Table S2: All strains and plasmids used in this study.**

| **Strains** | | |
| --- | --- | --- |
| **Strain name** | **Genotype** | **Source** |
| ***E. coli* strains** | | |
| DH5α | *F-φ80lacZΔM15* Δ(*lacZYA-argF*)*U169 recA1 endA1 hsdR17*(*rk−, mk+*) *phoA supE44 thi-1 gyrA96 relA1 λ−* | Invitrogen |
| BL21-CodonPlus-RIL | *F– ompT hsdS* (*rB – mB –* ) *dcm+ Tetr gal endA Hte* [*argU ileY leuW Camr* ] | New England Biolabs |
| BTH101 | Bacterial 2-hybrid strain | Euromedex |
| SM10 | *thi-1 thr leu tonA lacY supE recA*::*RP4-2-Tc::Mu* (*KmR*) | Invitrogen |
| PilS/PilS | BTH101 with PilS in pUT18C and pKT25 vectors | (67) |
| T18/T25 | BTH101 with empty pUT18C and pKT25 vectors | This study |
| FimX/FimX | BTH101 with FimX in pUT18C and pKT25 vectors | This study |
| FimX AAA/FimX AAA | BTH101 with FimX AAA in pUT18C and pKT25 vectors | This study |
| FimX-T18/FimX AAA-T25 | BTH101 with FimX in pUT18C and FimX AAA in pKT25 vectors | This study |
| FimX-T25/FimX AAA-T18 | BTH101 with FimX AAA in pUT18C and FimX in pKT25 vectors | This study |
| FimX-T18/PilZ-T25 | BTH101 with FimX in pUT18C and PilZ in pKT25 vectors | This study |
| FimX-T25/PilZ-T18 | BTH101 with PilZ in pUT18C and FimX in pKT25 vectors | This study |
| FimX AAA-T18/PilZ-T25 | BTH101 with FimX AAA in pUT18C and PilZ in pKT25 vectors | This study |
| FimX AAA-T25/PilZ-T18 | BTH101 with PilZ in pUT18C and FimX AAA in pKT25 vectors | This study |
| ***P. aeruginosa* strains** | | |
| mPAO1 | WT | (74) |
| *pilA*::Tn5 | mPAO1 *pilA* transposon mutant | (74) |
| Δ*fimX* | mPAO1 *fimX* deletion | This study |
| FimX AAA | mPAO1 FimX EVL motif to AAA mutation | This study |
| Δ*pilZ* | mPAO1 *pilZ* deletion | This study |
| Δ*fimX pilZ* | mPAO1 *fimX pilZ* double deletion | This study |
| Δ*fimX pilB* Δ1bp | mPAO1 *fimX* deletion with *pilB* Δ1bp suppressor | This study |
| Δ*fimX* PilB T430P | mPAO1 *fimX* deletion with PilB T430P suppressor | This study |
| Δ*fimX* PilO P168L | mPAO1 *fimX* deletion with PilO P168L suppressor | This study |
| Δ*fimX* PilJ T750I | mPAO1 *fimX* deletion with PilJ T570I suppressor | This study |
| Δ*fimX* FimL L115F | mPAO1 *fimX* deletion with FimL L115F suppressor | This study |
| Δ*fimX* CpdA V258G | mPAO1 *fimX* deletion with CpdA V258G suppressor | This study |
| mPAO1 + pMS402-*pcdrA*:LuX | mPAO1 with cdGMP reporter *pcdrA*:LuX cassette in pMS402 | This study |
| *pilA*::Tn5 + pMS402-*pcdrA*:LuX | *pilA*::Tn5 with cdGMP reporter *pcdrA*:LuX cassette | This study |
| mPAO1 + pMS402 | mPAO1 with empty pMS402 | This study |
| mPAO1 + pMS402-*pcdrA*:LuX pBADGr-*sadC* | mPAO1 with cdGMP reporter *pcdrA*:LuX cassette in pMS402 and *sadC* in pBADGr | This study |
| Δ*fimX* + pMS402-*pcdrA*:LuX | Δ*fimX* with cdGMP reporter *pcdrA*:LuX cassette in pMS402 | This study |
| Δ*fimX pilB* Δ1bp + pMS402-*pcdrA*:LuX | Δ*fimX pilB* Δ1bp with cdGMP reporter *pcdrA*:LuX cassette in pMS402 | This study |
| Δ*fimX* PilB T430P + pMS402-*pcdrA*:LuX | Δ*fimX* PilB T430P with cdGMP reporter *pcdrA*:LuX cassette in pMS402 | This study |
| Δ*fimX* PilO P168L + pMS402-*pcdrA*:LuX | Δ*fimX* PilO P168L with cdGMP reporter *pcdrA*:LuX cassette in pMS402 | This study |
| Δ*fimX* PilJ T750I + pMS402-*pcdrA*:LuX | Δ*fimX* PilJ T570I with cdGMP reporter *pcdrA*:LuX cassette in pMS402 | This study |
| Δ*fimX* FimL L115F + pMS402-*pcdrA*:LuX | Δ*fimX* L115F with cdGMP reporter *pcdrA*:LuX cassette in pMS402 | This study |
| Δ*fimX* CpdA V258G + pMS402-*pcdrA*:LuX | Δ*fimX* CpdA V258Gwith cdGMP reporter *pcdrA*:LuX cassette in pMS402 | This study |
| mPAO1 + pBADGr | mPAO1 with empty pBADGr | This study |
| mPAO1 + pBADGr-*sadC* | mPAO1 with *sadC* in pBADGr | This study |
| mPAO1 + pBADGr-*ydeH* | mPAO1 with *ydeH* in pBADGr | This study |
| Δ*fimX* + pBADGr | Δ*fimX* with empty pBADGr | This study |
| Δ*fimX* + pBADGr-*sadC* | Δ*fimX* with *sadC* in pBADGr | This study |
| Δ*fimX* + pBADGr-*ydeH* | Δ*fimX* with *ydeH* in pBADGr | This study |
| mPAO1 + pBADGr CyaB R456L | mPAO1 with CyaB R456L in pBADGr | (42) |
| mPAO1 + cAMP reporter | mPAO1 with cAMP reporter plasmid | This study |
| *pilA*::Tn5 + cAMP reporter | *pilA*::Tn5 with cAMP reporter plasmid | This study |
| Δ*cyaB* + cAMP reporter | Δ*cyaB* with cAMP reporter plasmid | This study |
| mPAO1 + cAMP reporter pBADGr-CyaB R456L | mPAO1 with cAMP reporter plasmid and CyaB R456L in pBADGr | This study |
| Δ*fimX* + cAMP reporter | Δ*fimX* with cAMP reporter plasmid | This study |
| Δ*fimX pilB* Δ1bp + cAMP reporter | Δ*fimX pilB* Δ1bp with cAMP reporter plasmid | This study |
| Δ*fimX* PilB T430P + cAMP reporter | Δ*fimX* PilB T430P with cAMP reporter plasmid | This study |
| Δ*fimX* PilO P168L + cAMP reporter | Δ*fimX* PilO P168L with cAMP reporter plasmid | This study |
| Δ*fimX* PilJ T750I + cAMP reporter | Δ*fimX* PilJ T570I with cAMP reporter plasmid | This study |
| Δ*fimX* FimL L115F + cAMP reporter | Δ*fimX* FimL L115F with cAMP reporter plasmid | This study |
| Δ*fimX* CpdA V258G + cAMP reporter | Δ*fimX* CpdA V258G with cAMP reporter plasmid | This study |
| mPAO1 + pHERD30T | mPAO1 with empty pHERD30T | This study |
| *pilA*::Tn5 + pHERD30T | *pilA*::Tn5 with empty pHERD30T | This study |
| Δ*fimX* + pHERD30T | Δ*fimX* with empty pHERD30T | This study |
| Δ*fimX pilB* Δ1bp + pHERD30T | Δ*fimX pilB* Δ1bp with empty pHERD30T | This study |
| Δ*fimX* PilB T430P + pHERD30T | Δ*fimX* PilB T430P with empty pHERD30T | This study |
| Δ*fimX* PilO P168L + pHERD30T | Δ*fimX* PilO P168L with empty pHERD30T | This study |
| Δ*fimX* PilJ T750I + pHERD30T | Δ*fimX* PilJ T570I with empty pHERD30T | This study |
| Δ*fimX* FimL L115F + pHERD30T | Δ*fimX* FimL L115F with empty pHERD30T | This study |
| Δ*fimX* CpdA V258G + pHERD30T | Δ*fimX* CpdA V258G with empty pHERD30T | This study |
| mPAO1 + pHERD30T-*fimX* | mPAO1 with *fimX* in pHERD30T | This study |
| *pilA*::Tn5 + pHERD30T-*fimX* | *pilA*::Tn5 with *fimX* in pHERD30T | This study |
| Δ*fimX* + pHERD30T-*fimX* | Δ*fimX* with *fimX* in pHERD30T | This study |
| Δ*fimX pilB* Δ1bp + pHERD30T-*fimX* | Δ*fimX pilB* Δ1bp with *fimX* in pHERD30T | This study |
| Δ*fimX* PilB T430P + pHERD30T-*fimX* | Δ*fimX* PilB T430P with *fimX* in pHERD30T | This study |
| Δ*fimX* PilO P168L + pHERD30T-*fimX* | Δ*fimX* PilO P168L with *fimX* in pHERD30T | This study |
| Δ*fimX* PilJ T750I + pHERD30T-*fimX* | Δ*fimX* PilJ T570I with *fimX* in pHERD30T | This study |
| Δ*fimX* FimL L115F + pHERD30T-*fimX* | Δ*fimX* FimL L115F with *fimX* in pHERD30T | This study |
| Δ*fimX* CpdA V258G + pHERD30T-*fimX* | Δ*fimX* CpdA V258G with *fimX* in pHERD30T | This study |
| mPAO1 + pHERD30T-FimX AAA | mPAO1 with FimX AAA pHERD30T | This study |
| *pilA*::Tn5 + pHERD30T-FimX AAA | *pilA*::Tn5 with FimX AAA pHERD30T | This study |
| Δ*fimX* + pHERD30T-FimX AAA | Δ*fimX* with FimX AAA pHERD30T | This study |
| Δ*fimX pilB* Δ1bp + pHERD30T-FimX AAA | Δ*fimX pilB* Δ1bp with FimX AAA pHERD30T | This study |
| Δ*fimX* PilB T430P + pHERD30T-FimX AAA | Δ*fimX* PilB T430P with FimX AAA pHERD30T | This study |
| Δ*fimX* PilO P168L + pHERD30T-FimX AAA | Δ*fimX* PilO P168L with FimX AAA pHERD30T | This study |
| Δ*fimX* PilJ T750I + pHERD30T-FimX AAA | Δ*fimX* PilJ T570I with FimX AAA pHERD30T | This study |
| Δ*fimX* FimL L115F + pHERD30T-FimX AAA | Δ*fimX* FimL L115F with FimX AAA pHERD30T | This study |
| Δ*fimX* CpdA V258G + pHERD30T-FimX AAA | Δ*fimX* CpdA V258G with FimX AAA pHERD30T | This study |
| Δ*fimX* + pHERD30T-His_6_-CyaB | Δ*fimX* with His_6_-CyaB in pHERD30T | This study |
| Δ*fimX* + pHERD30T-His_6_-CyaB D234S | Δ*fimX* with His_6_-CyaB D234S in pHERD30T | This study |
| Δ*fimX* Δ*cyaB* + pHERD30T | Δ*fimX* Δ*cyaB* with empty pHERD30T | This study |
| Δ*cyaB* + pHERD30T | Δ*cyaB* with empty pHERD30T | This study |
| Δ*cyaB* + pHERD30T-His_6_-CyaB | Δ*cyaB* with His_6_-CyaB in pHERD30T | This study |
| Δ*cyaB* + pHERD30T-His_6_-CyaB D234S | Δ*cyaB* with His_6_-CyaB D234S in pHERD30T | This study |
| Δ*pilG pilH* + pHERD30T | Δ*pilG pilH* with empty pHERD30T | This study |
| Δ*pilG pilH* + pHERD30T-His_6_-CyaB | Δ*pilG pilH* with His_6_-CyaB in pHERD30T | This study |
| Δ*pilG pilH* + pHERD30T-His_6_-CyaB D234S | Δ*pilG pilH* with His_6_-CyaB D234S in pHERD30T | This study |
| Δ*vfr* | mPAO1 *vfr* deletion | (20) |
| *pilO*::FRT | mPAO1 *pilO* FRT insertion | (37) |
| mPAO1 *pvfr*:mRUBY3 | mPAO1 with mRUBY3 cassette inserted upstream of the *pilMNOPQ pvfr* promoter | This study |
| *pilA*::Tn5 *pvfr*:mRUBY3 | *pilA*::Tn5 with mRUBY3 cassette inserted upstream of the *pilMNOPQ pvfr* promoter | This study |
| Δ*fimX pvfr*:mRUBY3 | Δ*fimX* with mRUBY3 cassette inserted upstream of the *pilMNOPQ pvfr* promoter | This study |
| Δ*vfr pvfr*:mRUBY3 | Δ*vfr* with mRUBY3 cassette inserted upstream of the *pilMNOPQ pvfr* promoter | This study |
| Δ*cyaB pvfr*:mRUBY3 | Δ*cyaB* with mRUBY3 cassette inserted upstream of the *pilMNOPQ pvfr* promoter | This study |
| mPAO1 *pvfr*:mRUBY3 + pBADGr R456L | mPAO1 with mRUBY3 cassette inserted upstream of the *pilMNOPQ pvfr* promoter and CyaB R456L in pBADGr | This study |
| Δ*fimX pilB* Δ1bp *pvfr*:mRUBY3 | Δ*fimX* *pilB* Δ1bp with mRUBY3 cassette inserted upstream of the *pilMNOPQ pvfr* promoter | This study |
| Δ*fimX* PilB T430P *pvfr*:mRUBY3 | Δ*fimX* PilB T430P with mRUBY3 cassette inserted upstream of the *pilMNOPQ pvfr* promoter | This study |
| Δ*fimX* PilO P168L *pvfr*:mRUBY3 | Δ*fimX* PilO P168L with mRUBY3 cassette inserted upstream of the *pilMNOPQ pvfr* promoter | This study |
| Δ*fimX* PilJ T750I *pvfr*:mRUBY3 | Δ*fimX* PilJ T570I with mRUBY3 cassette inserted upstream of the *pilMNOPQ pvfr* promoter | This study |
| Δ*fimX* FimL L115F *pvfr*:mRUBY3 | Δ*fimX* FimL L115F with mRUBY3 cassette inserted upstream of the *pilMNOPQ pvfr* promoter | This study |
| Δ*fimX* CpdA V258G *pvfr*:mRUBY3 | Δ*fimX* CpdA V258G with mRUBY3 cassette inserted upstream of the *pilMNOPQ pvfr* promoter | This study |
| Δ*xcpT* | mPAO1 *xcpT* deletion | Burrows lab |
| Δ*fimX pilB*::Tn5 + pHERD30T | Δ*fimX pilB*::Tn5 with empty pHERD30T | This study |
| Δ*fimX pilB*::Tn5 + pHERD30T-*pilB* | Δ*fimX pilB*::Tn5 with *pilB* in pHERD30T | This study |
| Δ*fimX pilB*::Tn5 + pHERD30T-*pilB* Δ1bp | Δ*fimX pilB*::Tn5 with *pilB* Δ1bp in pHERD30T | This study |
| Δ*fimX pilB*::Tn5 + pHERD30T-*pilB* Δ1bp-3 | Δ*fimX pilB*::Tn5 with *pilB* Δ1bp-3 in pHERD30T | This study |
| Δ*fimX pilB*::Tn5 + pHERD30T-*pilB* Δ1bp-6 | Δ*fimX pilB*::Tn5 with *pilB* Δ1bp-6 in pHERD30T | This study |
| Δ*fimX pilB*::Tn5 + pHERD30T-*pilB* Δ1bp-9 | Δ*fimX pilB*::Tn5 with *pilB* Δ1bp-9 in pHERD30T | This study |
| Δ*fimX pilB*::Tn5 + pHERD30T-*pilB* Δ1bp-12 | Δ*fimX pilB*::Tn5 with *pilB* Δ1bp-12 in pHERD30T | This study |
| Δ*fimX pilB*::Tn5 + pHERD30T-*pilB* Δ1bp-15 | Δ*fimX pilB*::Tn5 with *pilB* Δ1bp-15 in pHERD30T | This study |
| Δ*fimX pilB*::Tn5 + pHERD30T-*pilB* Δ1bp-18 | Δ*fimX pilB*::Tn5 with *pilB* Δ1bp-18 in pHERD30T | This study |
| Δ*fimX pilB*::Tn5 + pHERD30T-*pilB* Δ1bp-21 | Δ*fimX pilB*::Tn5 with *pilB* Δ1bp-21 in pHERD30T | This study |
| *pilB*::Tn5 | mPAO1 *pilB* transposon mutant | (74) |
| *pilB*::Tn5 + pHERD30T | *pilB*::Tn5 with empty pHERD30T | This study |
| *pilB*::Tn5 + pHERD30T-*pilB* | *pilB*::Tn5 with *pilB* in pHERD30T | This study |
| *pilB*::Tn5 + pHERD30T-*pilB* Δ1bp | *pilB*::Tn5 with *pilB* Δ1bp in pHERD30T | This study |
| *pilB*::Tn5 + pHERD30T-*pilB* Δ1bp-3 | *pilB*::Tn5 with *pilB* Δ1bp-3 in pHERD30T | This study |
| *pilB*::Tn5 + pHERD30T-*pilB* Δ1bp-6 | *pilB*::Tn5 with *pilB* Δ1bp-6 in pHERD30T | This study |
| *pilB*::Tn5 + pHERD30T-*pilB* Δ1bp-9 | *pilB*::Tn5 with *pilB* Δ1bp-9 in pHERD30T | This study |
| *pilB*::Tn5 + pHERD30T-*pilB* Δ1bp-12 | *pilB*::Tn5 with *pilB* Δ1bp-12 in pHERD30T | This study |
| *pilB*::Tn5 + pHERD30T-*pilB* Δ1bp-15 | *pilB*::Tn5 with *pilB* Δ1bp-15 in pHERD30T | This study |
| *pilB*::Tn5 + pHERD30T-*pilB* Δ1bp-18 | *pilB*::Tn5 with *pilB* Δ1bp-18 in pHERD30T | This study |
| *pilB*::Tn5 + pHERD30T-*pilB* Δ1bp-21 | *pilB*::Tn5 with *pilB* Δ1bp-21 in pHERD30T | This study |
| mPAO1 + pHERD30T-*pilB* | mPAO1 with *pilB* in pHERD30T | This study |
| mPAO1 + pHERD30T-*pilB* Δ1bp | mPAO1 with *pilB* Δ1bp in pHERD30T | This study |
| mPAO1 + pHERD30T-*pilB* Δ1bp-3 | mPAO1 with *pilB* Δ1bp-3 in pHERD30T | This study |
| mPAO1 + pHERD30T-*pilB* Δ1bp-6 | mPAO1 with *pilB* Δ1bp-6 in pHERD30T | This study |
| mPAO1 + pHERD30T-*pilB* Δ1bp-9 | mPAO1 with *pilB* Δ1bp-9 in pHERD30T | This study |
| mPAO1 + pHERD30T-*pilB* Δ1bp-12 | mPAO1 with *pilB* Δ1bp-12 in pHERD30T | This study |
| mPAO1 + pHERD30T-*pilB* Δ1bp-15 | mPAO1 with *pilB* Δ1bp-15 in pHERD30T | This study |
| mPAO1 + pHERD30T-*pilB* Δ1bp-18 | mPAO1 with *pilB* Δ1bp-18 in pHERD30T | This study |
| mPAO1 + pHERD30T-*pilB* Δ1bp-21 | mPAO1 with *pilB* Δ1bp-21 in pHERD30T | This study |
| mPAO1 PilB D566V | PilB D566V chromosomal knock-in | This study |
| mPAO1 PilB D566K | PilB D566K chromosomal knock-in | This study |
| mPAO1 PilB D566E | PilB D566E chromosomal knock-in | This study |
| mPAO1 PilB D566^STOP^ | PilB D566 to STOP codon chromosomal knock-in | This study |
| Δ*fimX pilB*::Tn5 + PilB T430P | Δ*fimX pilB*::Tn5 with PilB T430P in pHERD30T | This study |
| Δ*fimX pilB*::Tn5 + PilB T430S | Δ*fimX pilB*::Tn5 with PilB T430S in pHERD30T | This study |
| Δ*fimX pilB*::Tn5 + PilB T430N | Δ*fimX pilB*::Tn5 with PilB T430N in pHERD30T | This study |
| Δ*fimX pilB*::Tn5 + PilB T430I | Δ*fimX pilB*::Tn5 with PilB T430I in pHERD30T | This study |
| Δ*fimX pilB*::Tn5 + PilB L429P | Δ*fimX pilB*::Tn5 with PilB L429P in pHERD30T | This study |
| Δ*fimX pilB*::Tn5 + PilB R431P | Δ*fimX pilB*::Tn5 with PilB R431P in pHERD30T | This study |
| Δ*fimX pilB*::Tn5 + PilB R431A | Δ*fimX pilB*::Tn5 with PilB R431A in pHERD30T | This study |
| *pilB*::Tn5 + PilB T430P | *pilB*::Tn5 with PilB T430P in pHERD30T | This study |
| *pilB*::Tn5 + PilB T430S | *pilB*::Tn5 with PilB T430S in pHERD30T | This study |
| *pilB*::Tn5 + PilB T430N | *pilB*::Tn5 with PilB T430N in pHERD30T | This study |
| *pilB*::Tn5 + PilB T430I | *pilB*::Tn5 with PilB T430I in pHERD30T | This study |
| *pilB*::Tn5 + PilB L429P | *pilB*::Tn5 with PilB L429P in pHERD30T | This study |
| *pilB*::Tn5 + PilB R431P | *pilB*::Tn5 with PilB R431P in pHERD30T | This study |
| *pilB*::Tn5 + PilB R431A | Δ*pilB*::Tn5 with PilB R431A in pHERD30T | This study |
| mPAO1 + PilB T430P | mPAO1 with PilB T430P in pHERD30T | This study |
| mPAO1 + PilB T430S | mPAO1with PilB T430S in pHERD30T | This study |
| mPAO1 + PilB T430N | mPAO1Tn5 with PilB T430N in pHERD30T | This study |
| mPAO1 + PilB T430I | mPAO1 with PilB T430I in pHERD30T | This study |
| mPAO1 + PilB L429P | mPAO1 with PilB L429P in pHERD30T | This study |
| mPAO1 + PilB R431P | mPAO1 with PilB R431P in pHERD30T | This study |
| *pilB*::Tn5 + PilB R431A | mPAO1 with PilB R431A in pHERD30T | This study |
| *pilB* Δ1bp | mPAO1 *pilB* Δ1bp chromosomal knock-in | This study |
| PilB T430P | mPAO1 PilB T430P chromosomal knock-in | This study |
| *pilB* Δ1bp FimX AAA | *pilB* Δ1bp FimX AAA chromosomal knock-in | This study |
| PilB T430P FimX AAA | PilB T430P FimX AAA chromosomal knock-in | This study |
| PilA A86C | PilA A86C chromosomal knock-in | This study |
| Δ*fimX* PilA A86C | Δ*fimX* PilA A86C chromosomal knock-in | This study |
| Δ*fimX pilB* Δ1bp PilA A86C | Δ*fimX* *pilB* Δ1bp PilA A86C chromosomal knock-in | This study |
| *pilB* Δ1bp PilA A86C | *pilB* Δ1bp PilA A86C chromosomal knock-in | This study |
| Δ*fimX* CpdA V258G PilA A86C | Δ*fimX* CpdA V258G PilA A86C chromosomal knock-in | This study |
| Δ*pilT* | mPAO1 *pilT* deletion | This study |
| PilA A86C Δ*pilT* | PilA A86C chromosomal knock-in and *pilT* deletion | This study |
| Δ*fimX* PilA A86C Δ*pilT* | Δ*fimX* PilA A86C chromosomal knock-in and *pilT* deletion | This study |
| Δ*fimX pilB* Δ1bp PilA A86C Δ*pilT* | Δ*fimX* *pilB* Δ1bp PilA A86C chromosomal knock-in and *pilT* deletion | This study |
| *pilB* Δ1bp PilA A86C Δ*pilT* | *pilB* Δ1bp PilA A86C chromosomal knock-in and *pilT* deletion | This study |
| Δ*fimX* CpdA V258G PilA A86C Δ*pilT* | Δ*fimX* CpdA V258G PilA A86C chromosomal knock-in and *pilT* deletion | This study |
| Δ*fimX* + pHERD30T-mNGr-FimX | Δ*fimX* with FimX N-terminally tagged with mNeonGreen in pHERD30T | This study |
| Δ*fimX pilB*::Tn5 + pHERD30T-mNGr-FimX | Δ*fimX pilB*::Tn5 with FimX N-terminally tagged with mNeonGreen in pHERD30T | This study |
| Δ*fimX* + pHERD30T-mNGr-FimX AAA | Δ*fimX* with FimX AAA N-terminally tagged with mNeonGreen in pHERD30T | This study |
| Δ*fimX pilB*::Tn5 + pHERD30T-mNGr-FimX AAA | Δ*fimX pilB*::Tn5 with FimX AAA N-terminally tagged with mNeonGreen in pHERD30T | This study |
| **Plasmid list** | | |
| **Plasmid name** | **Characteristics** | **Source** |
| pEX18Gm | Suicide vector for gene replacement | (66) |
| pEX18Gm-Δ*fimX* | *fimX* deletion construct | This study |
| pEX18Gm-Δ*pilZ* | *pilZ* deletion construct | This study |
| pEX18Gm-Δ*cyaB* | *cyaB* deletion construct | This study |
| pEX18Gm-*pilB* Δ1bp | *pilB* Δ1bp (A1697) deletion construct | This study |
| pEX18Gm-PilB T430P | PilB T430P chromosomal knock-in construct | This study |
| pEX18Gm-CpdA V258G | CpdA V258G chromosomal knock-in construct | This study |
| pEX18Gm- Δ*pilG pilH* | *pilG* and *pilH* double deletion construct | This study |
| pEX18Gm-*pvfr*-mRUBY3 | mRUBY3 knock-in construct upstream of *pilMNOPQ* *vfr* promoter | This study |
| pEX18Gm-PilB D566V | PilB D566V chromosomal knock-in construct | This study |
| pEX18Gm-PilB D566K | PilB D566K chromosomal knock-in construct | This study |
| pEX18Gm-PilB D566E | PilB D566E chromosomal knock-in construct | This study |
| pEX18Gm-PilB D566^STOP^ | PilB D566 STOP codon chromosomal knock-in construct | This study |
| pEX18Gm-FimX AAA | FimX EVL motif to AAA chromosomal knock-in construct | This study |
| pEX18Gm-PilA A86C | PilA A86C chromosomal knock-in construct | This study |
| pEX18Gm-Δ*pilT* | *pilT* deletion construct | Burrows lab |
| pHERD30T | Broad host-range expression vector | (75) |
| pHERD30T-*fimX* | *fimX* expression construct | This study |
| pHERD30T-FimX AAA | FimX AAA expression construct | This study |
| pHERD30T-His_6_-CyaB | His_6_-CyaB expression construct | This study |
| pHERD30T-His_6_-CyaB D234S | His_6_-CyaB D234S expression construct | This study |
| pHERD30T-*pilB* | PilB expression construct | This study |
| pHERD30T-*pilB* Δ1bp | *pilB* Δ1bp expression construct | This study |
| pHERD30T-*pilB* Δ1bp-3 | *pilB* Δ1bp with one codon deleted from the 3ʹ end expression construct | This study |
| pHERD30T-*pilB* Δ1bp-6 | *pilB* Δ1bp with two codons deleted from the 3ʹ end expression construct | This study |
| pHERD30T-*pilB* Δ1bp-9 | *pilB* Δ1bp with three codons deleted from the 3ʹ end expression construct | This study |
| pHERD30T-*pilB* Δ1bp-12 | *pilB* Δ1bp with four codons deleted from the 3ʹ end expression construct | This study |
| pHERD30T-*pilB* Δ1bp-15 | *pilB* Δ1bp with five codons deleted from the 3ʹ end expression construct | This study |
| pHERD30T-*pilB* Δ1bp-18 | *pilB* Δ1bp with six codons deleted from the 3ʹ end expression construct | This study |
| pHERD30T-*pilB* Δ1bp-21 | *pilB* Δ1bp with seven codons deleted from the 3ʹ end expression construct | This study |
| pHERD30T-PilB T430P | PilB T430P expression construct | This study |
| pHERD30T-PilB T430S | PilB T430S expression construct | This study |
| pHERD30T-PilB T430N | PilB T430N expression construct | This study |
| pHERD30T-PilB T430I | PilB T430I expression construct | This study |
| pHERD30T-PilB L429P | PilB L429P expression construct | This study |
| pHERD30T-PilB R431P | PilB R431P expression construct | This study |
| pHERD30T-PilB R431A | PilB R431A expression construct | This study |
| pHERD30T-mNGr-FimX | FimX with N-terminal mNeonGreen fusion expression construct | This study |
| pHERD30T-mNGr-FimX AAA | FimX AAA with N-terminal mNeonGreen fusion expression construct | This study |
| pBADGr | Broad host-range expression vector | (5) |
| pBADGr-*sadC* | *sadC* expression construct | (20) |
| pBADGr-*ydeH* | *ydeH* expression construct | Howell lab |
| pBADGr-CyaB R456L | CyaB R456L expression construct | (42) |
| pMS402 | Broad host-range expression vector | (20) |
| pMS402-*pcdrA*:LuX | cdGMP reporter construct | (20) |
| cAMP reporter (*PaQa*:YFP/*rpoD*:mRUBY3) | cAMP reporter plasmid | (39) |
| pET28b-ΔN1D-His_6_-PilB | N-terminally truncated His_6_-tagged PilB for heterologous expression in *E. coli* | This study |
| pET28b-ΔN1D-His_6_-PilB K332A | N-terminally truncated His_6_-tagged PilB K332A for heterologous expression in *E. coli* | This study |
| pET28b-ΔN1D-His_6_-*PilB* Δ1bp | N-terminally truncated His_6_-tagged *pilB* Δ1bp for heterologous expression in *E. coli* | This study |
| pET28b-ΔN1D-His_6_-PilB T430P | N-terminally truncated His_6_-tagged PilB T430P for heterologous expression in *E. coli* | This study |
| pET28b-His_6_-FimX | His_6_-tagged FimX for heterologous expression in *E. coli* | This study |
