# Supplementary material for: Twitching motility suppressors reveal a role for FimX in type IV pilus extension dynamics
Source: PLoS Genet. 2025 Oct 13;21(10):e1011802. doi: 10.1371/journal.pgen.1011802 (PMC12533971; doi:10.1371/journal.pgen.1011802)
Supplement: S3 Table — (DOCX) [file pgen.1011802.s018.docx]

**Table S3: Primers used in this study.**

| **Primers** | | |
| --- | --- | --- |
| **Primer name** | **Characteristics** | **Sequence** |
| ΔFimX UpS_Fwd | For making delta FimX construct - Upstream region | ATATGAGCTCACGCAGATGGGCATCGAG |
| ΔFimX UpS_Rev | For making delta FimX construct - Upstream region | ATATTCTAGAATGTACGCGGGTCGCGT |
| ΔFimX DnS_Fwd | For making delta FimX construct - Downstream region | ATATTCTAGAGAGAGCGCCAGCGTCCTC |
| ΔFimX DnS_Rev | For making delta FimX construct - Downstream region | ATATAAGCTTCAACGAGTTGCAGATCGTCGAC |
| ΔPilZ UpS_Fwd | For making delta PilZ construct - Upstream region | ATATGAATTCGAGGCCCTGGAGGAACTGTT |
| ΔPilZ UpS_Rev | For making delta PilZ construct - Upstream region | ATATTCTAGACAGATTGGGTGGCAAACTCAT |
| ΔPilZ DnS_Fwd | For making delta PilZ construct - Downstream region | ATATTCTAGAAGTTCAACGACGGTGACAACAC |
| ΔPilZ DnS_Rev | For making delta PilZ construct - Downstream region | ATATAAGCTTGTGCACGATCACTGGCTTG |
| cpdA UpS_fwd | For making delta CpdA construct - Upstream region | ATATGAATTCAAGGGGAGCGGCTGCTC |
| cpdA UpS_rev | For making delta CpdA construct - Upstream region | ATATTCTAGATGGCTGTCCGAGAGCTGC |
| cpdA DnS_fwd | For making delta CpdA construct - Downstream region | ATATTCTAGACCTGGAAACGGGGATCTCG |
| cpdA DnS_rev | For making delta CpdA construct - Downstream region | ATATAAGCTTGTGGTCCTCGGTGAGTTCCC |
| cpdA V258G_fwd | For making V258G knockin construct when amplified from ΔfimX HT genomic template - pairs with cpdA DnS_rev primer | ATATGAATTCGGTTCGCCGGTAACCACG |
| FimX pHERD_Fwd | For cloning PAO1 FimX into pHERD | ATATTCTAGACTGAGCCCTTTCCATGG |
| FimX pHERD_Rev | For cloning PAO1 FimX into pHERD | ATATAAGCTTTCATTCGTCTCCCGAGG |
| PilZ pHERD_Fwd | For cloning PAO1 PilZ into pHERD | ATATGAATTCGGCAGGAACCTGCATGA |
| PilZ pHERD_Rev | For cloning PAO1 PilZ into pHERD | ATATAAGCTTTTACATCGTGTGGGTCGG |
| CpdA pHERD_Fwd | For cloning PAO1 cpdA into pHERD | ATATGAATTCAGGAGACGGCCCCTTG |
| CpdA pHERD_Rev | For cloning PAO1 cpdA into pHERD | ATATAAGCTTTCAGTATCCGGCGGTGT |
| CyaB UpS | For cloning CyaB upstream region | ATATGAATTCGAGTTCTACCCCTACTACCTGCAG |
| CyaB UpS_Rev | For cloning CyaB upstream region | ATATTCTAGACACGCGCGAATAGTATTCAC |
| CyaB DnS_Fwd | For cloning CyaB downstream region | ATATTCTAGAACTACGACAAGGAACGGGTC |
| CyaB DnS_Rev | For cloning CyaB downstream region | ATATAAGCTTAAAAGAACCTGGAGGCGTTC |
| FimX AAA_Fwd | For introducing AAA at the FimX EVL motif - has NotI cut site | GCCACGAGAACTACGCGGCCGCCCTGCGCCTGCTCAAT |
| FimX AAA_Rev | For introducing AAA at the FimX EVL motif - has NotI cut site | ATTGAGCAGGCGCAGGGCGGCCGCGTAGTTCTCGTGGC |
| PilB pHERD_Fwd | For cloning PilB into pHERD30T | ATATGAGCTCGCGATTCCTTCCCCATGA |
| PilB WT pHERD_Rev | For cloning wild-type PilB into pHERD30T | ATATTCTAGATTAATCCTTGGTCACGCGG |
| PilB d1bp pHERD | For cloning pilB delta 1bp into pHERD30T: pairs with PilB pHERD_Fwd | ATATTCTAGATTAACGCTTTGTCCGCCAT |
| PilB d1bp pHERD-3 | For cloning pilB delta 1bp into pHERD30T: pairs with PilB pHERD_Fwd | ATATTCTAGATTACTTTGTCCGCCATGGATTAAC |
| PilB d1bp pHERD-6 | For cloning pilB delta 1bp into pHERD30T: pairs with PilB pHERD_Fwd | ATATTCTAGATTATGTCCGCCATGGATTAACCT |
| PilB d1bp pHERD-9 New | For cloning pilB delta 1bp into pHERD30T: pairs with PilB pHERD_Fwd | ATATTCTAGATTACCGCCATGGATTAACCTTG |
| PilB d1bp pHERD-12 New | For cloning pilB delta 1bp into pHERD30T: pairs with PilB pHERD_Fwd | ATATTCTAGATTACCATGGATTAACCTTGGTCACG |
| PilB d1bp pHERD-15 New | For cloning pilB delta 1bp into pHERD30T: pairs with PilB pHERD_Fwd | ATATTCTAGATTATGGATTAACCTTGGTCACGC |
| PilB d1bp pHERD-18 New | For cloning pilB delta 1bp into pHERD30T: pairs with PilB pHERD_Fwd | ATATTCTAGATTAATTAACCTTGGTCACGCGGTT |
| PilB d1bp pHERD-21 New | For cloning pilB delta 1bp into pHERD30T: pairs with PilB pHERD_Fwd | ATATTCTAGATTAAACCTTGGTCACGCGGTT |
| PilM UpS-vfr rp_F | For cloning PilM upstream region into pEX18Gm to make vfr promoter mRUBY3 reporter | ATATGAATTCGCATTAGGCTTTTCACATCGAC |
| PilM UpS-vfr rp_R2 | For cloning PilM upstream region into pEX18Gm to make vfr promoter mRUBY3 reporter | ATATGGTACCTTCCCTATTAGCGTTCAATACTTACG |
| mRUBY3-vfr rp_F New2 | For cloning mRUBY3 into pEX18Gm to make vfr promoter reporter | ATATGGTACCATGGTGTCTAAGGGCGAAGAGC |
| mRUBY3-vfr rp_R New | For cloning mRUBY3 into pEX18Gm to make vfr promoter reporter | ATATTCTAGATTACTTGTACAGCTCGTCCATGCC |
| PilM DnS-vfr rp_F | For cloning PilM downstream region into pEX18Gm to make vfr promoter mRUBY3 reporter | ATATTCTAGAGTGCTAGGGCTCATAAAGAAGAAAG |
| PilM DnS-vfr rp_R | For cloning PilM downstream region into pEX18Gm to make vfr promoter mRUBY3 reporter | ATATAAGCTTCTGCTCAGCAGCGCATAG |
| CyaB D234S_F | For mutating CyaB D234 to S (has XhoI cut site): Pairs with CyaB pHERD_Rev | ACCGTGTTCTTCTCGAGCATCCGCGGCTTCACCGAG |
| CyaB D234S_R | For mutating CyaB D234 to S (has XhoI cut site): Pairs with CyaB pHERD_Fwd | CTCGGTGAAGCCGCGGATGCTCGAGAAGAACACGGT |
| CyaB His_Fwd | For cloning CyaB with N-terminal His-tag into pHERD30T - encodes his tag | ATATGAATTCATGCACCACCACCACCACCACATGAAGCCTACCCTCCCCG |
| CyaB His_Rev | For cloning CyaB with N-terminal His-tag into pHERD30T | ATATAAGCTTTTAGAGGATGACCTTGTCGCG |
| PilB N2D/CTD_Fwd | Encodes Pa PilB N2D and CTD with ΔD180-N terminus truncation for cloning into pET28b : Pairs with: Pa PilB_Rev | ATATGCTAGCGACGCACCTGTAGTACGTTTCGTC |
| Pa PilB_Rev | For cloning PilB into pET28b | ATATGAATTCTTAATCCTTGGTCACGCGGTT |
| Pa PilB d1bp pET28_Rev | For cloning PilB d1bp into pET28b. Pairs with PilB N2D/CTD_Fwd | ATATGAATTCTTAACGCTTTGTCCGCCATG |
| Pa PilB K332A_Fwd | For mutating PilB Walker A motif K332 to A | CCCACCGGCTCGGGCGCGACGGTATCGCTATACACC |
| Pa PilB K332A_Rev | For mutating PilB Walker A motif K332 to A | GGTGTATAGCGATACCGTCGCGCCCGAGCCGGTGGG |
| PilB T430P_Fwd | For knocking in PilB T430 to P forward mutagenesis primer | GCCGCCGAGACCCTGCCCCGGTTGCTGAACATGG |
| PilB T430P_Rev | For knocking in PilB T430 to P reverse mutagenesis primer | CCATGTTCAGCAACCGGGGCAGGGTCTCGGCGGC |
| PilB T430N_F | For mutating PilB T430 to N forward primer | AGCGCCGCCGAGACCCTGAACCGGTTGCTGAACATGGGC |
| PilB T430N_R | For mutating PilB T430 to N reverse primer | GCCCATGTTCAGCAACCGGTTCAGGGTCTCGGCGGCGCT |
| PilB T430S_F | For mutating PilB T430 to S forward primer | AGCGCCGCCGAGACCCTGTCCCGGTTGCTGAACATGGGC |
| PilB T430S_R | For mutating PilB T430 to S reverse primer | GCCCATGTTCAGCAACCGGGACAGGGTCTCGGCGGCGCT |
| PilB T430I_F | For mutating PilB T430 to I forward primer | AGCGCCGCCGAGACCCTGATCCGGTTGCTGAACATGGGC |
| PilB T430I_R | For mutating PilB T430 to I reverse primer | GCCCATGTTCAGCAACCGGATCAGGGTCTCGGCGGCGCT |
| PilB R431A_F | For mutating PilB R431 to A forward primer | AGCGCCGCCGAGACCCTGACCGCGTTGCTGAACATGGGC |
| PilB R431A_R | For mutating PilB R431 to A reverse primer | GCCCATGTTCAGCAACGCGGTCAGGGTCTCGGCGGCGCT |
| PilB D566X UpS_Fwd | For knocking in PilB C-terminal residue upstream region: pairs with PilB D566X DnS_Rev | ATATGAATTCAGATCCGCGACCTGGAGAC |
| PilB D566X DnS_Rev | For knocking in PilB C-terminal residue upstream region: pairs with PilB D566X UpS_Fwd | ATATTCTAGACGATTCCGTTTTTTCCTTGTAGGT |
| PilB D566V_Fwd | For knocking in PilB D566 to V forward mutagenesis primer - removes AseI cut site | AACCGCGTGACCAAGGTTTAATCCATGGCGGACA |
| PilB D566V_Rev | For knocking in PilB D566 to V reverse mutagenesis primer - removes AseI cut site | TGTCCGCCATGGATTAAACCTTGGTCACGCGGTT |
| PilB D566K_Fwd | For knocking in PilB D566 to K forward mutagenesis primer - removes AseI cut site | AACCGCGTGACCAAGAAGTAATCCATGGCGGACA |
| PilB D566K_Rev | For knocking in PilB D566 to K reverse mutagenesis primer - removes AseI cut site | TGTCCGCCATGGATTACTTCTTGGTCACGCGGTT |
| PilB D566E_Fwd | For knocking in PilB D566 to E forward mutagenesis primer - removes AseI cut site | AACCGCGTGACCAAGGAGTAATCCATGGCGGACA |
| PilB D566E_Rev | For knocking in PilB D566 to E reverse mutagenesis primer - removes AseI cut site | TGTCCGCCATGGATTACTCCTTGGTCACGCGGTT |
| PilB D566STOP_Fwd | For knocking in PilB D566 to STOP codon forward mutagenesis primer - removes AseI cut site | AACCGCGTGACCAAGTAATAATCCATGGCGGACA |
| PilB D566STOP_Rev | For knocking in PilB D566 to STOP codon reverse mutagenesis primer - removes AseI cut site | TGTCCGCCATGGATTATTACTTGGTCACGCGGTT |
| PilB T430P UpS_Fwd | For knocking in PilB T430 to P forward mutagenesis primer - removes natural EcoRV cut site | ATATGAATTCGATATATCCGAACGACGCAAAC |
| PilB T430P DnS_Rev | For knocking in PilB T430 to P reverse mutagenesis primer | ATATTCTAGACTGCCCGGTCAGTCAGTTC |
| PilB T430P_Fwd | For knocking in PilB T430 to P forward mutagenesis primer | GCCGCCGAGACCCTGCCCCGGTTGCTGAACATGG |
| PilB T430P_Rev | For knocking in PilB T430 to P reverse mutagenesis primer | CCATGTTCAGCAACCGGGGCAGGGTCTCGGCGGC |
| FimX pET28_Fwd | For cloning FimX into pet28b | ATATGCTAGCATGGCCATCGAAAAGAAAACC |
| FimX pET28_Rev | For cloning FimX into pet28b | ATATAAGCTTTTATTCGTCTCCCGAGGAG |
| PilA A86C Fwd | For mutating PAO1 PilA A86C | GGCGTCGAGCCGGATTGTAACAAGTTGGGTGTA |
| PilA A86C Rev | For mutating PAO1 PilA A86C | TACACCCAACTTGTTACAATCCGGCTCGACGCC |
| PIlA A86 Ups_Fwd | PilA A86 primer for upstream region amplification | ATATGAATTCCGCTCAGTTGGATGCTGTC |
| PIlA A86 Dns_Rev | PilA A86 primer for downstream region amplification | ATATTCTAGAGCCAAGCTGGAAGCTTCC |
| PilG Ups_Fwd | For making a PilG and PilH double deletion | ATATGAGCTCTCCGCTTCCAGTTCGAAC |
| PilG Ups_Rev | For making a PilG and PilH double deletion | ATATTCTAGAGAATCGTTTTCGAATCGTCG |
| PilH Dns_Fwd | For making a PilG and PilH double deletion | ATATTCTAGATGGACGAAGAGACCCTGC |
| PilH Dns_Rev | For making a PilG and PilH double deletion | ATATAAGCTTAGCTGTTCGCTGAAGGTGTC |
| mNeonGreen-FimX_F1 | For making an mNeonGreen-FimX N-terminal fusion construct - has FimX RBS and adds ATG start codon | ATATTCTAGACTGAGCCCTTTCCATGGTGAGCAAGGGCGAGGAG |
| mNeonGreen-FimX_R1 | For making an mNeonGreen-FimX N-terminal fusion construct - encodes 5G linker | ACCACCACCACCACCCTTGTACAGCTCGTCCATGCC |
| mNeonGreen-FimX_F2 | For making an mNeonGreen-FimX N-terminal fusion construct - encodes 5G linker | GGTGGTGGTGGTGGTATGGCCATCGAAAAGAAAACC |
| mNeonGreen-FimX_R2 | For making an mNeonGreen-FimX N-terminal fusion construct | ATATAAGCTTTCATTCGTCTCCCGAGGAGA |
| PilB R431P_F | For mutating PilB R431 to P. Pairs with: PilB WT pHERD_Rev | AGCGCCGCCGAGACCCTGACCCCGTTGCTGAACATGGGC |
| PilB R431P_R | For mutating PilB R431 to P. Pairs with:PilB pHERD_Fwd | GCCCATGTTCAGCAACGGGGTCAGGGTCTCGGCGGCGCT |
| PilB L429P_F | For mutating PilB L429 to P. Pairs with: PilB WT pHERD_Rev | AGCGCCGCCGAGACCCCGACCCGGTTGCTGAACATGGGC |
| PilB L429P_R | For mutating PilB L429 to P. Pairs with:PilB pHERD_Fwd | GCCCATGTTCAGCAACCGGGTCGGGGTCTCGGCGGCGCT |
